# “Appropriateness” of Clinical Data Under Regulation (EU) 2017/745– A Case Study and Survey

**DOI:** 10.1007/s43441-025-00827-6

**Published:** 2025-07-03

**Authors:** Elisabeth Oltmanns, Michael D’Agosto, Folker Spitzenberger

**Affiliations:** 1 Escentia GmbH, Simeonscarré 2, 32423 Minden, Germany; 2 Technische Hochschule Lübeck, Mönkhofer Weg 239, 23562 Lübeck, Germany; 3 Hochschule Furtwangen University, Campus Tuttlingen, Kronenstraße 16, 78532 Tuttlingen, Germany; 4 Fraunhofer Research Institution for Individualized and Cell-Based Medical Engineering, Mönkhofer Weg 239a, 23562 Lübeck, Germany

**Keywords:** Medical devices, European medical device regulations, Clinical evaluation, Clinical data, Medium risk medical device

## Abstract

**Purpose:**

Regulation (EU) 2017/745, the European Medical Device Regulation (MDR), raises clinical evidence requirements but lacks clarity on what constitutes “sufficient clinical evidence” for medium-risk, Class IIb non-implantable CE-marked devices. This research investigates whether a clinical evaluation of a newly developed Class IIb device can be conducted without a clinical investigation and explores the role of data from the same generic device group in clinical evaluations.

**Methods:**

Expert interviews with notified body reviewers and a survey were conducted to assess the regulatory landscape and the appropriateness of non-clinical data.

**Results:**

Findings reveal inconsistencies in the interpretation of MDR among notified bodies. While some reviewers accepted clinical evaluations based on non-clinical data, others required clinical or equivalent device data. The exclusion of data from the same generic device group under MDR complicates compliance and may impose unnecessary burdens on manufacturers, particularly for standard-of-care devices with well-documented safety profiles. Survey results indicate discrepancies in the role of non-clinical data, with notified bodies favouring standard-based bench testing while manufacturers and consultants advocate for advanced testing methodologies, such as in silico models. The study also highlights differing perspectives on the role of post-market clinical follow-up (PMCF) in clinical evaluations.

**Conclusions:**

This research underscores the need for standardized guidance on clinical data requirements and the role of non-clinical evidence. Addressing these gaps is essential to balance patient safety with innovation and streamline the regulatory pathway for medium-risk medical devices, ensuring a more predictable and efficient approval process in the EU.

## Introduction

Regulation (EU) 2017/745, the European Medical Device Regulation (MDR) published in May 2017, was shaped predominantly by two medical device scandals that had occurred in previous years: the early failure and other adverse effects associated with metal-on-metal hip implants and the use of non-medical grade silicone in breast implants. As a result, the regulation placed an explicit focus on patient safety and health based on demonstrated clinical evidence for CE-marked medical devices according to the new regulation [1, 2].

Although clinical data was not explicitly required for medical devices which are not classified as Class III or implantable until the revision of the Medical Device Directive (MDD) in 2007 [3], the MDR, which came into full application in 2021, obligates manufacturers to base the “*confirmation of conformity with relevant general safety and performance requirements”* (MDR 61(1)) on *“clinical data providing sufficient clinical evidence”*.

According to Article 61(4–6), MDR further specifies the requirements to perform a clinical investigation for Class III and implantable devices while providing exclusions for devices for which equivalence can be shown with a previous version, devices previously marketed under MDD (legacy devices) and devices listed under MDR Article 61(6b) which are considered well-established technology. Additionally, the Medical Device Coordination Group (responsible for issuing guidance on the application of MDR, in the following referred to as MDCG) has offered additional information on the clinical data expectations for legacy devices (MDCG 2020-6) [4]. However, there remains a lack of a more detailed definition of sufficient clinical data or the appropriateness of clinical data for medium and low risks devices.

In a survey of medical device manufacturers on the challenges associated with the increased clinical evaluation requirements of the MDR, 80% of respondents identified the amount of data needed to generate “sufficient clinical evidence” as a significant challenge, regardless of whether the devices in question were classified as high-risk or medium-risk. Among those surveyed, 60% of respondents from the medium-risk device category indicated their intention to conduct a new clinical investigation [5].

However, while Kearney and McDermott [5] assert that post-market surveillance (PMS) data and literature reviews represent the most crucial sources of clinical data for medium-risk legacy devices (devices previously marketed under MDD), this is not a viable option for the initial CE marking of newly developed medical devices.

Considering the costs and timeline associated with a clinical investigation, which represents the sole method for generating clinical data from the use of a newly developed medical device prior to CE marking, it is imperative for manufacturers to gain a comprehensive understanding of the requirements for “sufficient clinical evidence” at the earliest stages of device development.

In particular, when considering standard-of-care devices with medium risk, where there is substantial clinical experience with the same generic device group but limited published data on specific devices, this type of investment may appear to be a questionable use of resources.

While there are uncertainties about the clinical data requirements, higher thresholds for the use of data from equivalent devices due to more precise criteria, and a lack of clinical data for lower risk devices, it becomes more common to bring medical devices to the market based solely on non-clinical data only as described in MDR Art. 61(10). These clinical evaluations rely on performance evaluation, bench testing and pre-clinical evaluation. In another study by Kearney and McDermott [6], clinical evaluations under this route were found to be particularly challenging due to a lack of clarity as to when this article could be applied.

This research seeks to address these regulatory challenges through expert interviews with notified body reviewers responsible for assessing clinical evaluations and by conducting a stakeholder survey. A hypothetical clinical strategy for a laparoscopic insufflator was developed and discussed with notified body representatives to clarify MDR interpretations concerning clinical data for newly developed medium-risk devices. Additionally, survey responses were analyzed to explore non-clinical data usage in clinical evaluations.

The study focused therefore on three research questions:

1. Is it still possible under MDR to perform a clinical evaluation of a newly developed class IIb non-implantable active medical device without performing a clinical investigation?

2. Which role does data from the same generic device group / the clinical state of the art play in this context?

3. Which factors determine the type of (clinical) data required to show safety, performance, and benefit-risk ratio of a medical device?

## Materials and Methods

### Expert Interviews

In order to gain insight into the present interpretation of the MDR by notified body reviewers and to address the research questions, a qualitative approach, specifically expert interviews, was deemed the most appropriate methodology. The interview process was conducted in accordance with the methodology outlined by Kaiser [7] for planning and conducting expert interviews.

As an interview guide, a clinical strategy for a new development of a hypothetical laparoscopic insufflator (class IIb according to Annex VIII, MDR) was developed. The strategy was designed to cover the following points to evaluate potential factors which may influence decisions regarding appropriateness of clinical data:

- Indirect clinical benefit, which in this case means the device itself does not have a benefit but it allows for the performance of a specific procedure, as well as three clinical benefits applicable to laparoscopic insufflators in general (reduced surgery time compared to gasless laparoscopy, reduced risks of embolism compared to insufflation with ambient air and reduced risk of hypothermia due to heating of the CO2).

- Summary of the State of the Art to show that the device is standard of care and the clinical safety of the generic device group is well known.

- Describing the device as well-established technology (WET) as defined in MDCG 2020-6.

- Using considerations on risks, intended clinical performance and indirect benefit to assess the level of clinical evidence.

- Referring to the level of evidence required for WET, even if the subject device is not considered WET.

- Description of the non-clinical testing to show that the interaction between the device and the human body can be simulated in a non-clinical setting.

- A plan for post-market clinical follow-up (active collection of clinical data after initial CE-marking, PMCF) activities, describing a survey in which *“data on the safety and performance of the device are collected on a patient-by-patient or procedural basis”* [8].

The clinical strategy was provided to the interview partners in advance of the interview, allowing them to familiarise themselves with it in advance of the discussion.

During the interview, a PowerPoint presentation was employed to illustrate various elements of the clinical strategy covering the following topics:

- The level of clinical data required.

- The applicability of MDCG 2020-6 in general and the definition of WET more specifically.

- The influence of risk, clinical performance, and clinical benefit on the level of clinical evidence required.

- The acceptability of data from the generic device group.

The experts selected for this study were notified body reviewers, as they are the parties responsible for determining whether the level of clinical evidence presented in a clinical evaluation is sufficient for the evaluation of a specific device.

After initially contacting the reviewers via either personal contact data or through general contact information of the notified bodies, eight interview partners representing eight notified bodies within four EU Member States participated in the interviews.

At the conclusion of each interview, the interviewer would ascertain whether all pertinent questions had been addressed. If any remained unresolved, the specific questions were posed.

All interviews were transcribed automatically through Microsoft Teams, and the interviewer subsequently summarized the answers to the pre-defined questions and shared the records with the interviewee to allow for clarifications.

### Survey

A survey on the use of non-clinical data for clinical evaluation has been conducted to understand what type of non-clinical data is currently used in clinical evaluation and what data is considered appropriate by those performing and reviewing clinical evaluations.

The survey aimed at manufacturers, Notified Bodies and consultants and has been distributed via LinkedIn, by the Bundesverband Medizintechnologie e.V. (BVMed) to its members and to notified body staff contacted for expert interviews as described above.

QuestionPro (https://www.questionpro.com) has been selected as the tool for the online survey.

The questionnaire included background information on job description, size of organization and experience with clinical evaluations and risk classes, followed by questions on the type of data used in clinical evaluations. Participants were asked to respond for each risk class in which they have experience and given the option to provide any additional information they consider helpful.

The first question (Q1) focuses on the type of data used for clinical evaluation in general.

The other two questions focus on the type of non-clinical data used, the first (Q2) asking participants which data they see in use and the second (Q3) asking which data they consider appropriate.

**Fig. 1:**
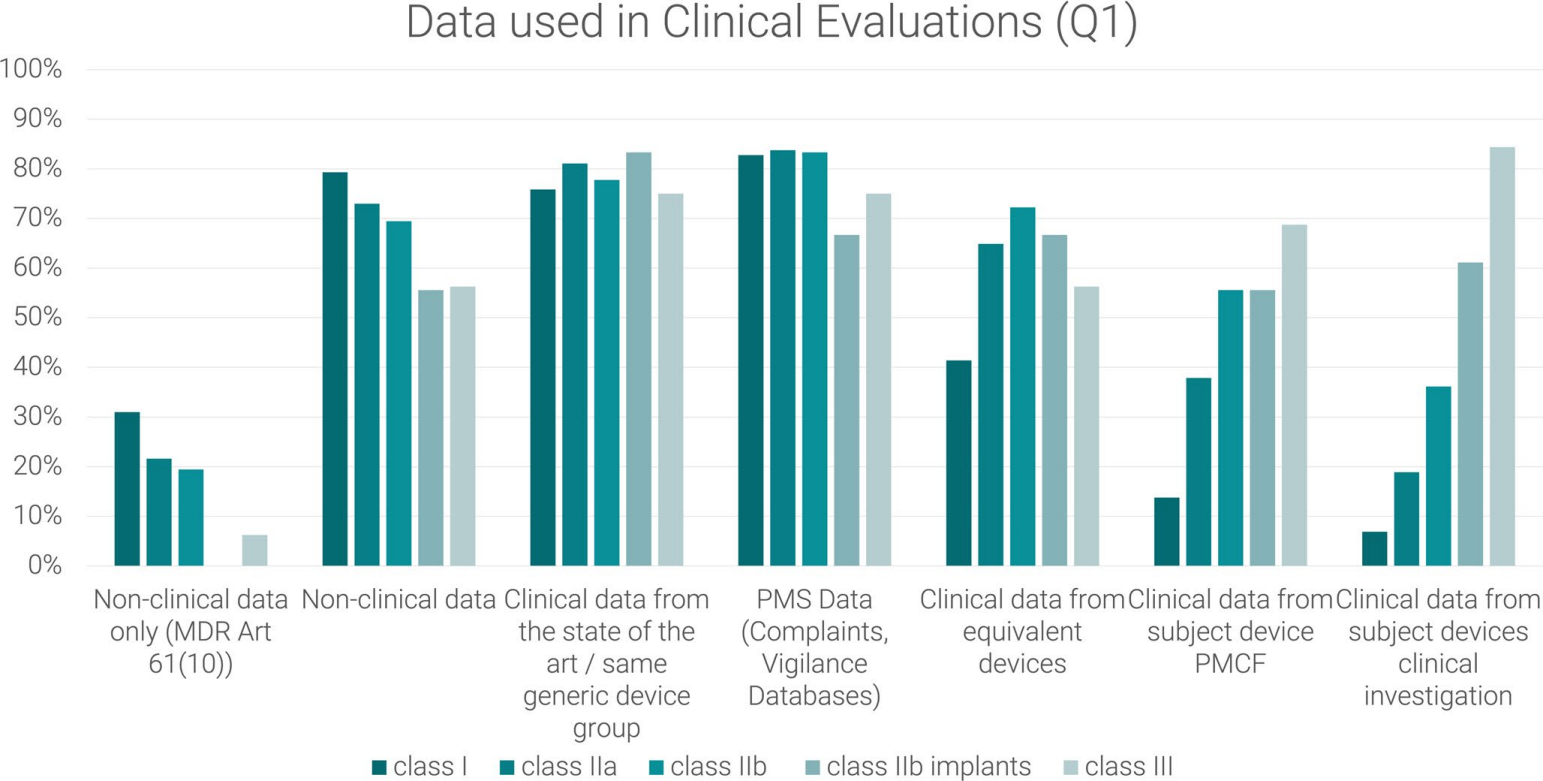
Observed use of data in clinical evaluations (Q1) as reported by medical device manufacturers, notified body employees and consultants: non-clinical data only refers to clinical evaluations where the conformity of the medical device with the relevant general safety and performance requirements was based purely on non-clinical data while non-clinical data could have been used supportively in any clinical evaluation. Clinical data from the medical State-of-the-Art and the generic device group is not considered clinical data under the MDR. Post-market surveillance (PMS) Data includes any complaints received by the manufacturer and data found in vigilance data bases while post-market clinical follow up includes clinical data from clinical studies, registries, surveys and other active measures

**Fig. 2:**
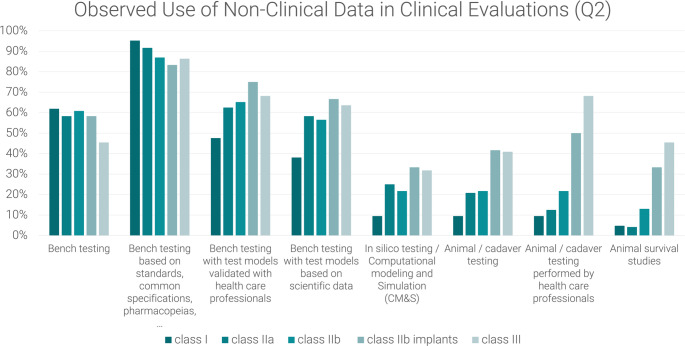
Observed use of non-clinical data in clinical evaluations (Q2) as reported by medical device manufacturers, notified body employees and consultants

**Fig. 3:**
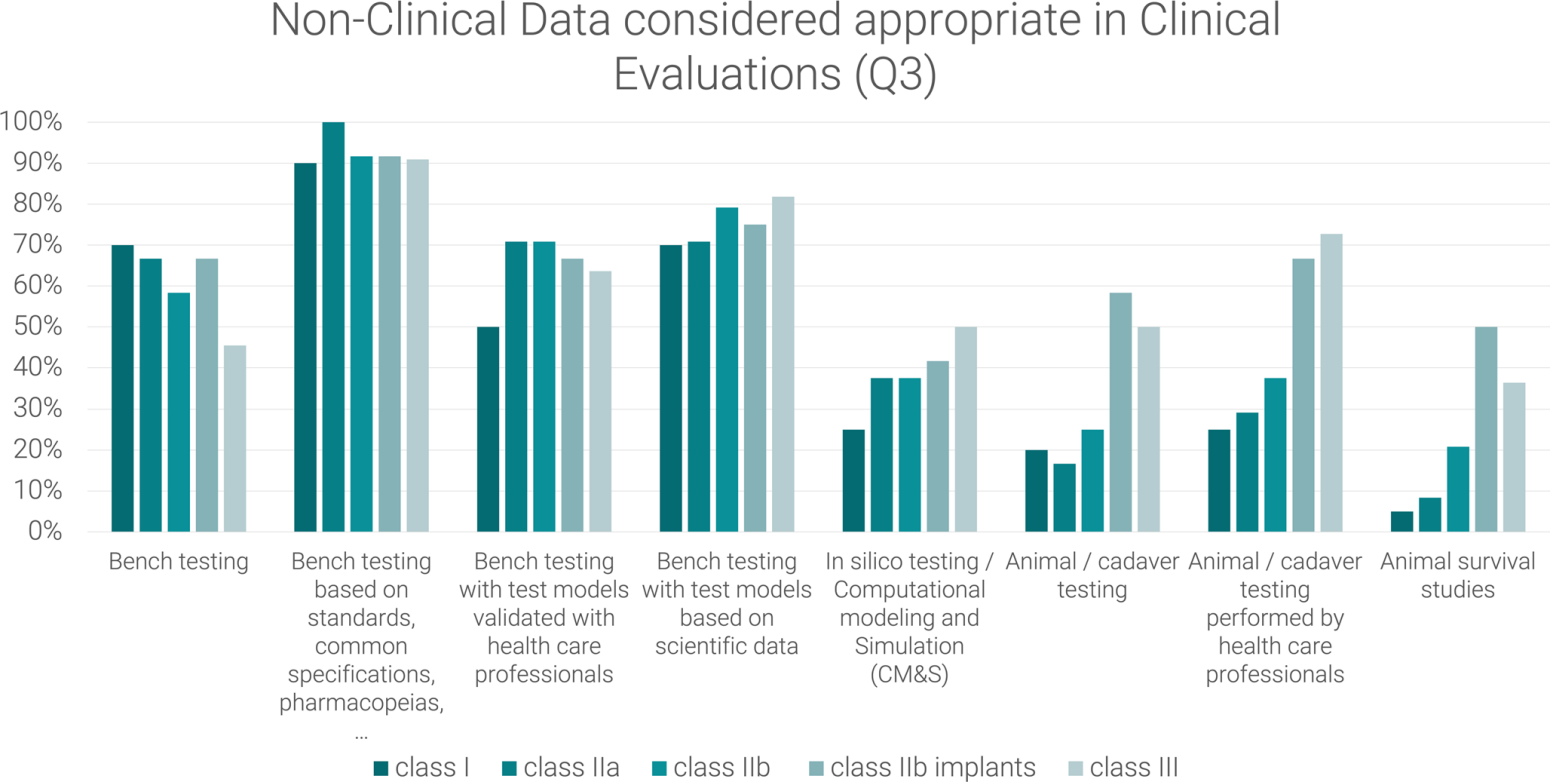
Non-clinical data considered appropriate in clinical evaluations (Q3) as reported by medical device manufacturers, notified body employees and consultants

The original wording of the questions was:

- Q1: Please select per risk class which data you see **predominantly used** in the clinical evaluation documentation to support safety, performance and benefit-risk profile (multiple answers possible, for possible answers refer to Fig. 1).
- Q2: Please select per risk class which non-clinical data you see **predominantly used** in the clinical evaluations to support safety, performance and benefit-risk profile (multiple answers possible, for possible answers refer to Fig. 2).
- Q3: Please select per risk class, which non-clinical data you personally– according to your experience - **consider appropriate** in the clinical evaluations to support safety, performance and benefit-risk profile (multiple answers possible, for possible answers refer to Fig. 3).

For each of the questions, the respondents were given the option to add any additional information they consider useful.

## Results

### Expert Interviews

Although it was initially planned to request all notified bodies designated under the EU MDR for an interview, the decision was taken not to contact any further notified bodies following the conclusion drawn from the first eight interviews with employees from eight distinct notified bodies from four different EU Member States. This decision was based on the results of the interviews, which indicated that there was no common understanding of how the MDR requirements should be applied to the subject device.

Two questions were integrated into the interview process: one regarding the utilization of data from equivalent devices, as this was a recurrent recommendation from interview participants, and another concerning the impact of the risk class on the determination of the requisite level of clinical evidence.

**Fig. 4:**
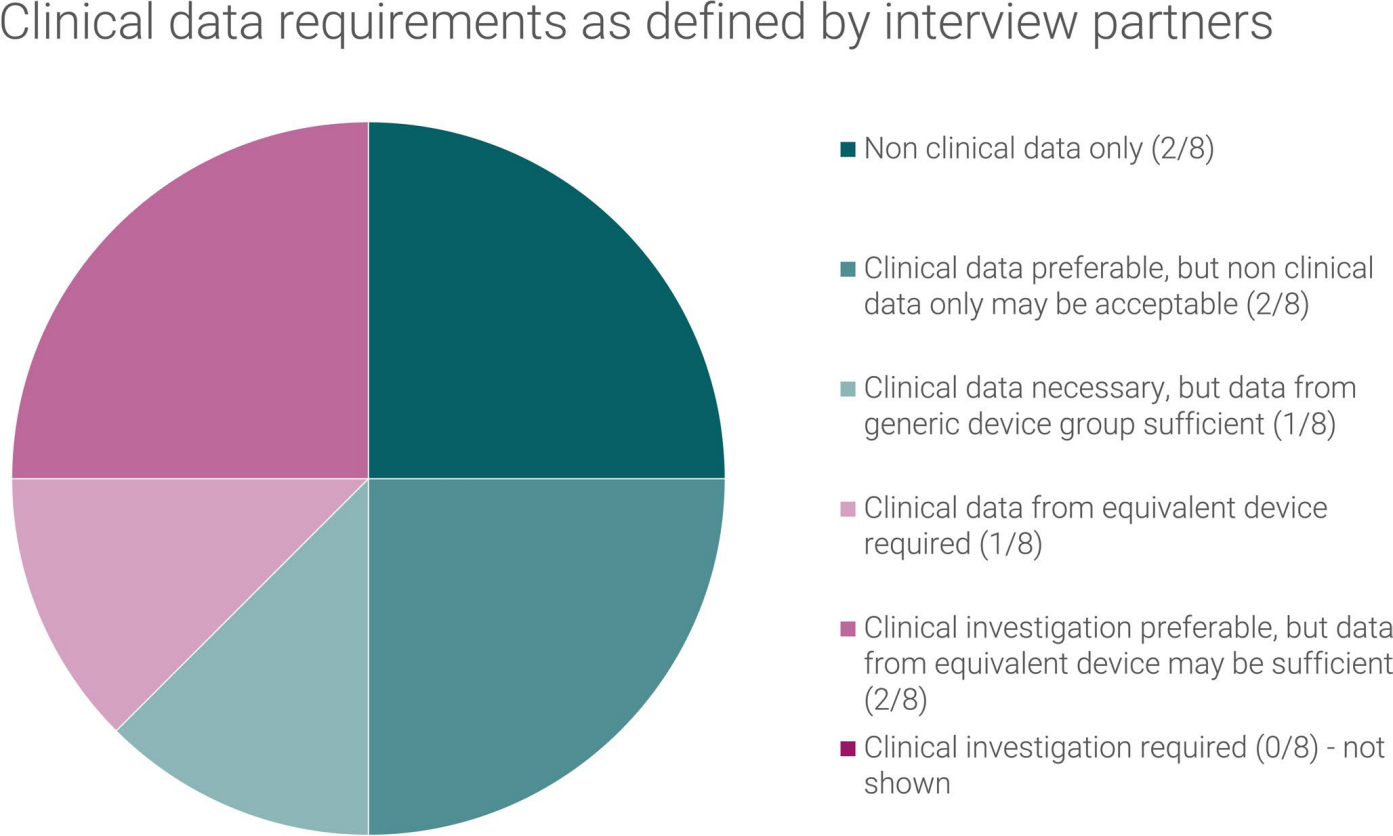
Clinical data requirements as defined by interview partners for the initial CE marking of the hypothetical laparoscopic insufflator

The clinical data requirements for the initial CE marking of the subject device, as defined by the interview partners, have been summarized in Fig. 4.

It should be noted for any discussed clinical strategy, that the description of the medical State-of-the-Art including data from the generic device group and benchmark devices was always considered.

Two of the interview partners asserted that the utilization of non-clinical data would be adequate for the subject device. Two of the interview partners indicated a preference for the inclusion of clinical data, although they also suggested that a clinical evaluation based solely on non-clinical data might be feasible in certain circumstances. One of the respondents articulated a slightly different perspective, proposing that although clinical data is required for the substantiation of safety and performance, and clinical data should be collected after initial CE-marking (PMCF), the clinical data utilized for the initial CE marking may be derived from the medical State-of-the-Art / the same generic device group. They acknowledged that this was difficult to argue under MDR. One interview partner expressed a clear preference for data from an equivalent device, while two others indicated a strong need for clinical data but suggested that data from an equivalent device might be sufficient in certain circumstances.

The data from the same generic device group does not meet the criteria for classification as clinical data in accordance with the MDR. Consequently, five respondents asserted that it can only be utilized as supplementary evidence. One of them emphasized that it constituted clinical data, but that it was an issue that it was not explicitly identified as such in the MDR. Another respondent indicated that it could be employed in accordance with Appendix 3 of MDCG 2020-6, which describes a suggested hierarchy of clinical evidence for confirmation of conformity with relevant general safety and performance requirements under the MDR and gives some indication which level of clinical evidence is appropriate for high-risks devices.

All respondents concurred that the device is not included on the MDR Article 61(6b) list, which delineates well-established technology.

The respondents offered divergent opinions regarding the definition provided by MDCG 2020-6. Half of the respondents indicated that the subject device exhibited the common features listed, while two respondents cited the absence of market history, one respondent cited the device’s complex design, and one respondent cited both factors as reasons why the subject device did not fulfil the definition. Two respondents indicated that this question was of no value to them, as they asserted that the MDR Article 61(6b) list is the exclusive repository of products that can be considered well-established technology. They reasoned that the MDR is the overarching legislation and that the MDCG can only clarify, but not undermine, the MDR.

The six interview partners with whom the applicability of MDCG 2020-6 was discussed concurred that it was only applicable to devices which have been previously marketed under MDD. One interviewee additionally posited that the general idea may be followed, given the absence of a specific guideline for new developments at the time of the interview.

**Fig. 5:**
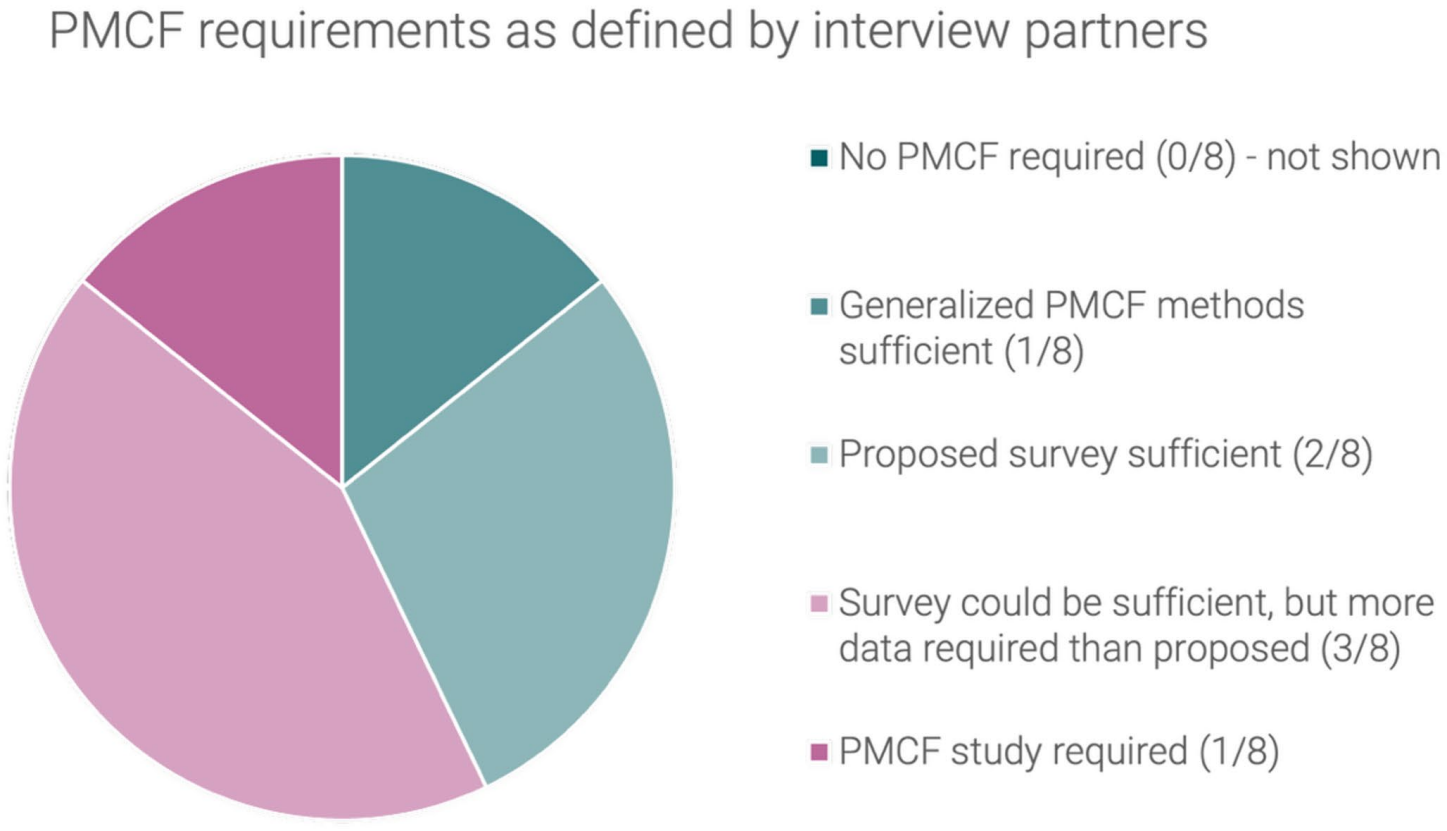
Requirements for post-market clinical follow-up (PMCF requirements) as defined by the interview partners for the initial CE marking of the hypothetical laparoscopic insufflator

The requirements for post-market clinical follow-up (PMCF requirements) for the initial CE marking of the subject device, as defined by the interview partners, can be summarized as follows (See Fig. 5):

One respondent asserted that a generalized PMCF would suffice, whereas another indicated that a PMCF study would be necessary in the absence of a pre-market clinical investigation. The remaining six interview partners, however, deemed a case-based survey to be an adequate PMCF measure for the subject device. However, three respondents emphasized the significance of statistical design and the necessity of including all pertinent patient populations.

**Fig. 6:**
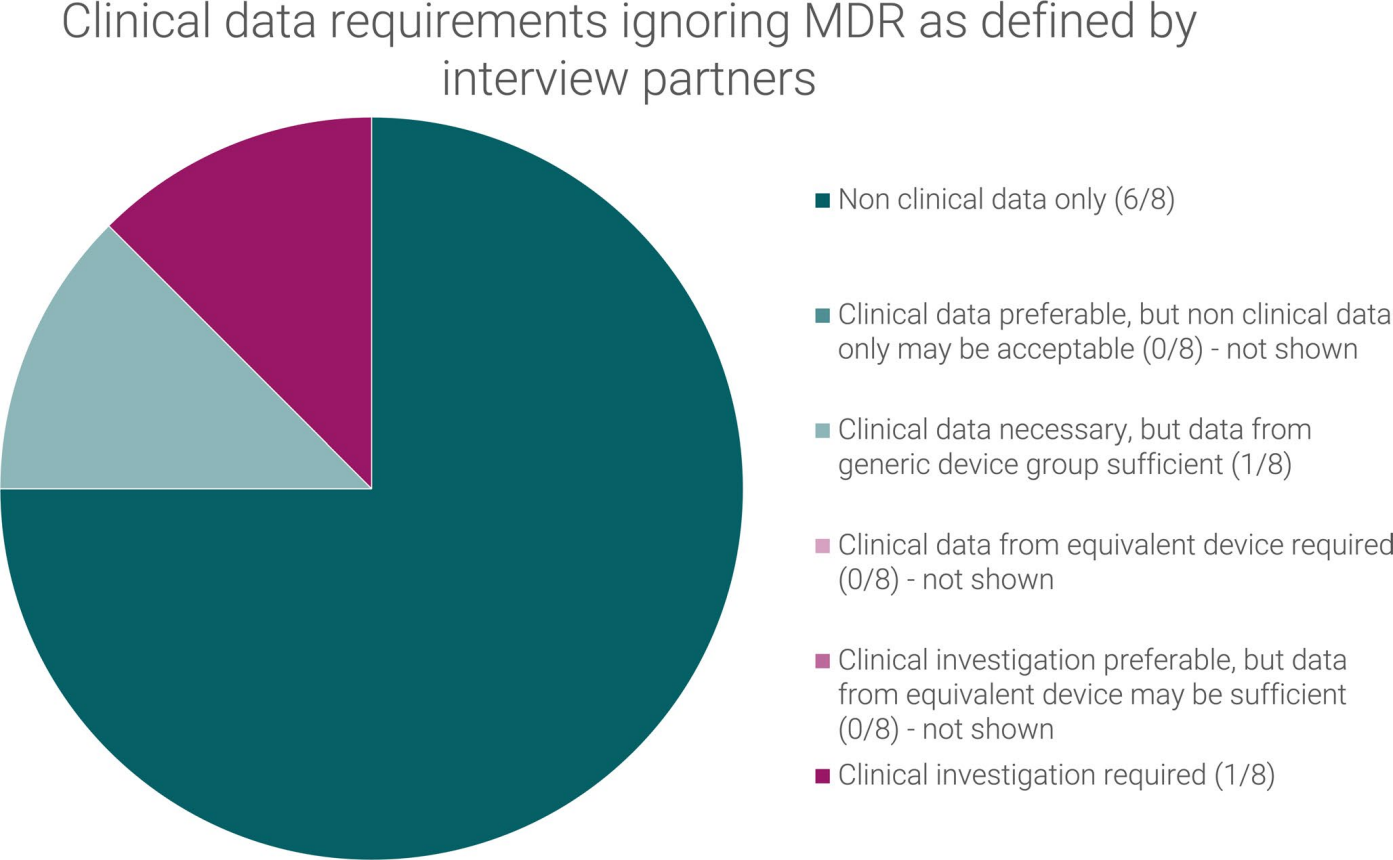
Clinical data requirements ignoring MDR and applying a common-sense approach as defined by interview partners for the initial CE marking of the hypothetical laparoscopic insufflator

The interview-partners were also asked which clinical data they would consider appropriate when ignoring the MDR and instead applying a common-sense approach based on their experience as a physician / veterinarian / biologist. The goal of this question was to understand to which extend their answers were shaped by their understanding of the MDR and whether they agreed with the MDR requirements for clinical data (See Fig. 6).

If the Medical Device Regulation (MDR) was not taken into consideration, only one respondent indicated a necessity for clinical data from the subject device. Another interview partner expressed a desire to see clinical data from an equivalent device, but without the MDR, they would consider the same generic device group equivalent. All other respondents deemed non-clinical data sufficient, citing the availability of data from the same generic device group and the necessity for new devices to demonstrate operation at the same settings.

### Survey

The survey was viewed by 244 participants, with 65 initiating the survey and 32 completing it. Among the 33 participants who did not complete the survey, 19 ceased participations during or after the background information section and 14 after the first set of main questions. Notably, all respondents who answered the second main question completed the survey.

Participants who had completed the first question were included in the analysis for Q1 but excluded from the subsequent questions.

The details on participant backgrounds are summarized in Table 1.

**Table 1:** Background of participants

	Q1	Q2 & Q3
Consultant	8	6
Medical Device Manufacturer	26	20
Notified Body	11	6
Other	1	0
Total	46	32

For all questions, multiple answers were possible, and the percentages were calculated by dividing the number of participants who claimed to have seen the data by the number of participants answering the question for the particular risk class. Consequently, the sum of the percentages does not equal 100%.

The two types of data that almost all respondents considered to be the main data used were clinical data from the state-of-the-art or generic device group and PMS data, including those from complaints and vigilance databases. The use of the data types was reported over all device group by 78% and 80% of the respondents respectively, with little variation between the different risk classes. All device groups used clinical data from equivalent devices and from the device PMCF. However, their observed use increased significantly with increased risk class. Of note, a high percentage of over 50% (Class III) to nearly 80% (Class I) of data in clinical evaluations was reported to be include non-clinical data (See Fig. 1).

Figure 2 visualises that the utilisation of bench testing based on standards, common specifications and pharmacopoeias appears to be the most prevalent non-clinical data in clinical evaluations across all risk classes, as evidenced by most respondents. The number of respondents observing the implementation of specific tests diminishes for lower risk classes, particularly as the complexity of the tests increases. Animal survival studies are predominantly observed for higher risk cases.

Bench testing based on more complex test models, either validated with health care professionals or based on scientific data, is less prevalent for class I (observed by less than 50% and less than 40% respectively) and predominantly observed for class IIb implants (over 70% and over 60% respectively), followed by class III products, as shown in Fig. 2. Interestingly, innovative computational modelling and simulation (CM&S) methods, also known as in silico trials, were not widely observed, but seem to get more relevance in higher risk products such as class IIb implants and class III products (more than 30%).

**Fig. 7:**
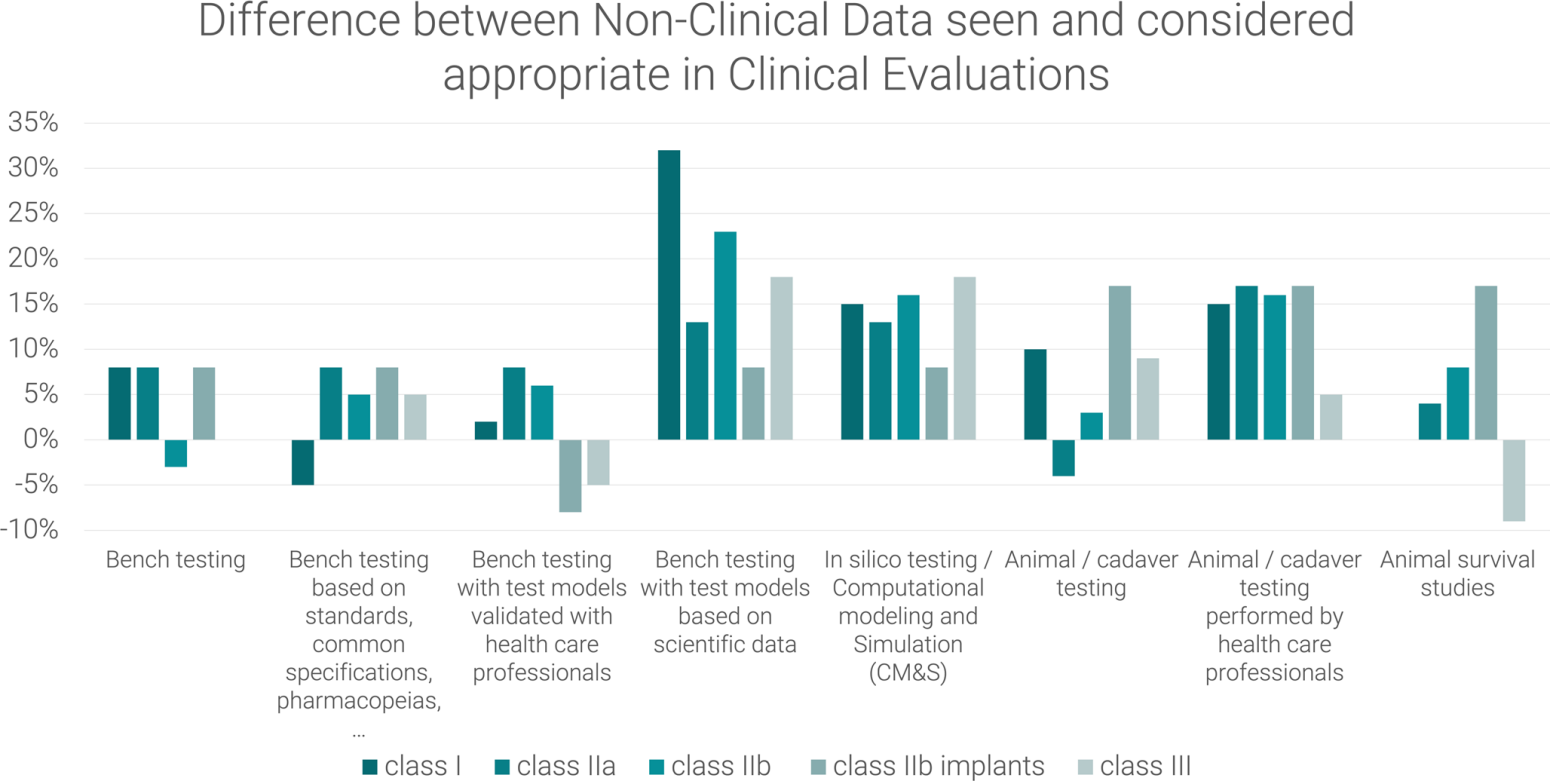
Difference between non-clinical data seen and considered appropriate in Clinical Evaluations as reported by medical device manufacturers, notified body employees and consultants. Values > 0 represent respondents who consider the data appropriate but do not see it, values < 0 represent respondents who see data but do not consider them appropriate.

An analysis of the responses to Q3 reveals the potential value of more complex bench testing, particularly that based on scientific data, as shown in Figs. 3 and 7. This form of testing is considered to be valuable for all risk classes, yet it appears to be underrepresented, particularly in class I devices. Over 30% respondents consider these tests appropriate but do not observe them.

The survey was completed by respondents from a variety of professional backgrounds, each of whom brings a unique perspective, particularly regarding the appropriateness of data. Consequently, the results were analysed by subgroup, acknowledging that this approach will lead to very small sample sizes.

**Fig. 8:**
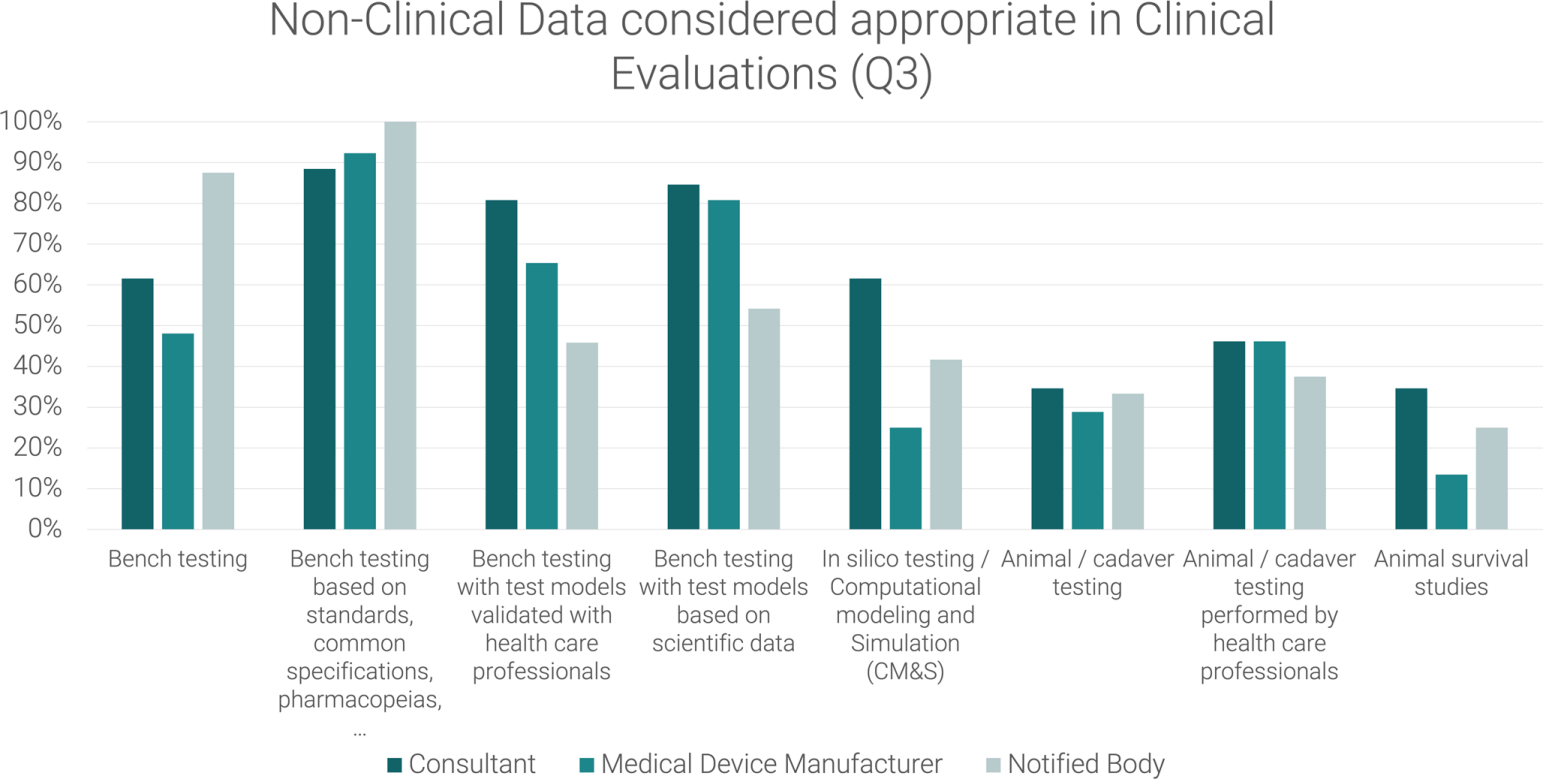
Non-Clinical data considered appropriate in clinical evaluations (Q3), Comparison between Consultant, Medical Device Manufacturer and Notified Body

**Fig. 9:**
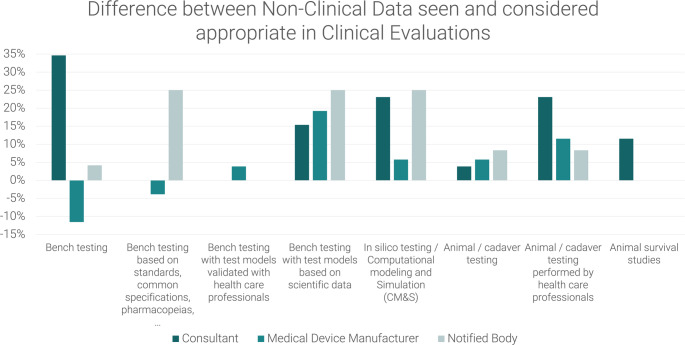
Difference between non-clinical data seen and considered appropriate in clinical evaluations–Comparison between Consultant, Medical Device Manufacturer and Notified Body Values > 0 represent respondents who consider the data appropriate but do not see it, values < 0 represent respondents who see data but do not consider them appropriate

A comparison of responses from respondents with different backgrounds, particularly with regard to the non-clinical data deemed appropriate, reveals a tendency among respondents from notified bodies to place greater reliance on general bench testing and testing based on standards, common specifications, and pharmacopoeias as visualised in Fig. 8. In contrast, they appear to attach less significance to bench tests with models validated by healthcare professionals or based on scientific data. It is noteworthy that a greater proportion of respondents regard these tests as appropriate, compared to those who currently observe their utilisation (Fig. 9).

With regard to unspecified bench testing, less than 50% of respondents from medical device manufacturers consider this appropriate, in contrast to the responses of nearly all notified bodies. Regarding innovative CM&S methods, the same tendency is observed (Fig. 8): While a greater number of notified bodies and consultants consider these methods to be appropriate (over 40% and over 60%, respectively), manufacturers seem to be more restrictive with the use of these methods and only around 20% estimate CM&S to be appropriate for use in clinical evaluations.

## Discussion

The central question guiding this research was as follows: Is it still possible under MDR to perform a clinical evaluation of a newly developed class IIb non-implantable active medical device without performing a clinical investigation?

The short answer to this question is yes – although clinical data may still be required. Through in-depth interviews with notified body representatives, it was ascertained that none of the respondents insisted on a clinical investigation for the subject device. However, while two of the eight respondents expressed willingness to accept a clinical evaluation based on non-clinical data, two others advocated for a clinical investigation, albeit with the caveat that data from an equivalent device may suffice, as per the MDR. It is noteworthy that when respondents were asked to disregard the MDR and exercise their discretion and medical expertise, only one respondent deemed a clinical investigation appropriate, while none of the others requested clinical data as defined by the MDR. This suggests that the stringent interpretation of MDR Article 61 by various stakeholders, rather than the fundamental need for clinical data to enhance patient safety, has contributed to heightened clinical data requirements in recent years.

The answer to the original research question is therefore dependent on the notified body or even the specific reviewer. This prompts the question of why different notified body reviewers reach such divergent interpretations of the same regulatory requirements and why following the MDR contradicts their expert opinion of what would be sufficient evidence for bringing this device to market.

### Appropriateness of Clinical Data (MDR)

An examination of the MDR (Article 61 and Article 2(48)) reveals that clinical data from the subject or an equivalent device is the default route for a clinical evaluation. Non-clinical data may only be used to support the safety and performance of medical devices if clinical data is deemed inappropriate. However, a thorough review of the MDR and MDCG documents reveals no explicit statement regarding specific circumstances under which clinical data is not required. To ascertain the factors that determine the type of (clinical) data required to demonstrate safety, performance and benefit-risk ratio of a medical device, the following should be consulted (MDR Art. 61(10)):

- Results of risk management.

- Interaction between the device and the human body.

- Intended clinical performance.

- Claims made by the manufacturer.

Despite the absence of official guidance on the matter, a webinar presented by Holborow (published by the notified body British Standards Institute, in the following referred to as BSI) offers further insights into the interpretation of a notified body. The webinar asserts that the exclusion of all devices that interact with the human body is not a prerequisite, and that basic surgical instruments are permitted under MDR Art. 61(10). However, it is emphasised that as soon as a clinical claim is made, clinical data must be provided [9].

This rather literal interpretation of the MDR described by BSI has been followed by all interview partners, although their interpretation of clinical claims and clinical benefit did differ.

Another factor resulting in different interpretations was the question of whether the post-market collection of clinical data was applicable for devices brought to market based on non-clinical data only. All interview partners allowing for a clinical evaluation based on non-clinical data only agreed to this route and were very clear that they required PMCF. This could be interpreted as an agreement that clinical data was not indispensable for initial CE marking, but that clinical data should be collected post-market. However, one interview partner excluded performing the clinical evaluation based on non-clinical data only for initial CE marking, citing the need for PMCF as one of the reasons.

### Data from the Same Generic Device Group

The utilisation of data from the generic device group was excluded by all but one interview participant. However, the availability of data may have influenced the decision-makers’ inclination to permit the exclusive use of non-clinical data.

This is a particularly noteworthy finding, as this factor is not mentioned in the MDR when considering the appropriateness of clinical data in Art. 61(10) and is further confirmed when examining MDR Art. 61 (1) which states that „that level of clinical evidence shall be appropriate in view of the characteristics of the device and its intended purpose.“.

The medical state of the art, which incorporates data from the generic device group and plays a pivotal role in clinical evaluations, is solely cited in the MDR as a source to ascertain the acceptability of the benefit-risk ratio. From this perspective, the information available from the generic device group holds no significance in determining appropriateness of clinical data. This approach, although consistent with MDR Art. 61(10), fails to differentiate between an incremental change of a standard of care device and a newly developed therapeutic approach.

Applying common sense to this question, the novelty of the device is undoubtedly one of the most significant factors: If the effects of CO_2_ insufflation on the human body were unknown and laparoscopic procedures had never been performed, it is highly improbable that a clinical evaluation based on non-clinical data would ever be considered for the device used as an example in the interviews.

This will also have been one of the more significant factors influencing the interview partners when they were asked to apply a common-sense approach to the question of clinical data: 7/8 respondents considered non-clinical data appropriate, especially when considering the knowledge gained from use of similar products of the last decades– the data from the generic device group.

The MDR facilitates the use of data from an equivalent device to substantiate the safety and performance of a novel development (MDR Art. 61(3a)). Nevertheless, the utilisation of data from a solitary device of equivalent function carries the risk of overlooking less prevalent risks or issues associated with alternative devices.

Consequently, the utilisation of data from the aforementioned generic device group, as opposed to a single equivalent device, appears to be the prevailing choice for standard-of-care devices.

Under MDD, particularly for lower-risk devices, any comparable device was deemed equivalent. Consequently, the data from the same generic device group was incorporated as clinical evidence for the performance and safety of the subject devices. However, the introduction of the MDR led to a complete cessation of this approach by establishing higher standards for equivalence (MDR Annex XIV 3, MDCG 2020-5) and reducing the role of data from the generic device group to the description of the medical State-of-the-Art. This resulted in the creation of benchmarks for specified performance parameters and qualitative and quantitative aspects of clinical safety.

MDCG 2020-6 appears to revive this “old” approach for devices of well-established technology: Adopting the concepts of MDCG 2020-6, which permit the utilisation of non-clinical data, such as data from the same generic device group, for devices falling within the MDCG 2020-6 definition of WET, would provide regulatory justification for information from the same generic device group to be considered in the assessment of the appropriateness of clinical data. However, this has been ruled out by the notified body interview partners who agreed that the concepts form MDCG 2020-6 could not be applied to new developments.

### Type of Non-Clinical Data

It appeared that the scientific approach to and representation of the human-body-device-interaction of the non-clinical data was not a contributing factor in the decision-making process of the interview partners. This suggests that the ability to adequately simulate the interaction between the device and the human body in a non-clinical setting to confirm the safety and performance of the device may not have been seen as a significant factor so far.

As evidenced by both the interviews and the survey, it appears that some notified body reviewers did not thoroughly examine the non-clinical data, as illustrated by their comments. Furthermore, 55% of notified body respondents only answered the question on overall data usage but not the questions on non-clinical data (drop-out rates for consultants and manufacturers were 25% and 23% respectively), and some of the interview partners avoided further discussion of non-clinical testing, focusing only on the question of specific standards.

While the type of non-clinical data does not seem to play a role when considering the appropriateness of clinical data in the MDR or the MDCG documents, it is crucial to consider the original intention of the MDR and to acknowledge the resources usually available for pre-clinical and clinical evaluation. The question therefore arises in how far high quality non-clinical data with a solid scientific approach and representation of the human-body-device-interaction could replace pre-market clinical data. In this context, it is noteworthy that initiatives in the EU and worldwide are increasingly focusing on the integration of innovative methodologies into the preclinical and clinical evaluation of medical devices [10, 11]. This includes procedures based on the 3R principle and the - at least partial - replacement of clinical trials by in silico trials [12, 13].

Central to these methods, however, is their credibility and a regulatory framework that allows the evidence obtained in this way to be appropriately incorporated into the medical device approval process. So far, the MDR has addressed CM&S methods only superficially and without specifying criteria for acceptance or credibility (MDR, Annex I, 10.1a and Annex VII, 4.5.4.a).

This approach, however, would necessitate the involvement of personnel with the appropriate qualifications to make such decisions, both from the manufacturer and the notified body.

### Limitations of this Research

In the interviews, potential bias may have influenced responses, as the interviewer typically advocates for manufacturers in regulatory submissions and may be inclined to minimise clinical data requirements. However, the use of a single interviewer ensured consistency. In addition, equivalent device data was excluded early due to limited availability, although most reviewers preferred its inclusion. Discussion of the required level of such data could have provided valuable insights for manufacturers, as it is comparable in resource requirements to non-clinical data collection.

The survey was designed to be concise to maintain a high response rate, but this brevity limited the depth of information gathered and relied on comment sections for further insight. Anonymity, while encouraging participation, prevented validation of responses. The small sample size further limited the ability to draw strong conclusions from subgroup analysis. In addition, the lack of standardised terminology for non-clinical testing made data interpretation difficult, as it was unclear whether all respondents understood the questionnaire in the same way. Despite these limitations, the results provide valuable perspectives on how MDR requirements are interpreted and highlight differing opinions on the role of non-clinical data in clinical evaluations.

### Impact of Current Developments

The importance and timeliness of this research is underlined by the number of initiatives currently underway to improve not only the understanding but also the application of the MDR, particularly in the area of clinical data.

However, most of them focus on the process of clinical evaluation (ISO 18969 and the MDCG guideline under development) or specifically on the list of high-risk devices of well-established technology referred to in MDR Art. 61(6b) [14, 15].

Only a single position paper issued by the European Association of Medical Devices Notified Bodies (Team NB) [16] addresses low and moderate risk devices and the application of MDR Art. 61(10). However, the proposal of specific criteria and a list of devices to which MDR Art. 61(10) can be applied without further research into where non-clinical data following a scientific approach to and representation of the human-body-device-interaction can replace clinical data could result in an undue burden on manufacturers to produce pre-market clinical data or rely on data from equivalent devices while failing to enhance patient safety.

Due to the factors currently applied to the adequacy of clinical data and the tendency of notified body assessors to err on the side of caution when requesting clinical data, such a list may be treated as a “not list” - with the result that any device not on this list will be excluded from the application of MDR Art. 61(10). Especially looking at the interviews conducted as part of this study, and the three out of eight interviewees who could not be convinced to apply MDR Art. 61(10) for the subject device, suggests that a list for which consensus is found may be very short.

## Conclusion

The findings of this research indicate that the interpretation of the MDR requirements with regard to the necessity of clinical investigations for the conduct a clinical evaluation varies significantly among notified bodies. This regulatory ambiguity presents substantial challenges for manufacturers seeking market approval for medium-risk medical devices in the EU.

While the regulation provides a structured framework for clinical evaluations, its application varies widely among notified bodies, creating uncertainty for manufacturers. The exclusion of data from the same generic device group and the lack of clear criteria for assessing the appropriateness of clinical data further complicate the regulatory landscape. Also, the potential of innovative methodologies like computational modelling & simulation and the criteria for their acceptance by regulatory bodies are currently underestimated and fragmentary, respectively.

The divergence in responses from the interview participants regarding the suitability of clinical data under the MDR, as compared to a common-sense evaluation of appropriateness, indicates that there is a fundamental need for a comprehensive revision of the understanding and potentially even the articulation of the requirements stipulated in MDR Art. 61.

Addressing these issues will require a concerted effort from regulators, notified bodies, and industry stakeholders to develop clear guidelines, standardize testing methodologies, and promote a more consistent approach to clinical evaluations. By doing so, the medical device industry can better navigate the regulatory environment while ensuring both patient safety and technological innovation.

In the interim, it is advisable for manufacturers to engage with their notified bodies to acquire a comprehensive understanding of their specific perspectives on this issue. Notwithstanding the exclusion of clinical strategy discussions from the structured dialogue as per the Team NB code of conduct, it remains feasible to incorporate this topic into discussions following the formalization of a contract with the Notified Body.

## Data Availability

No datasets were generated or analysed during the current study.
